# Oral health-related quality of life in diabetic patients: comparison of the Persian version of Geriatric Oral Health Assessment Index and Oral Health Impact Profile: A descriptive-analytic study

**DOI:** 10.1186/2251-6581-13-32

**Published:** 2014-02-04

**Authors:** Ava Nikbin, Mohammadali Bayani, Niloofar Jenabian, Soraya khafri, Mina Motallebnejad

**Affiliations:** 1 Faculty of Dentistry, Dentistry Student Research committee(DSRC), Babol University of Medical Sciences, Babol, Iran; 2 Department of Internal Medicine, Ayatollah Rouhani Hospital, Babol University of Medical Sciences, Babol, Iran; 3 Dental Materials Research Center ,Department of Periodontology & Implantology, Faculty of Dentistry, Babol University of Medical Sciences, Babol, Iran; 4 Department of Social Health Medicine, Babol University of Medical Sciences, Babol, Iran; 5 Cellular & Molecular Biology Research Center, Department of Oral Medicine, Faculty of Dentistry, Babol University of Medical Sciences, Babol, Iran

**Keywords:** Diabetes mellitus, Type 2, Oral health, Quality of Life, GOHAI, OHIP-14

## Abstract

**Background:**

Diabetes mellitus is one of the systemic disease which is show important oral manifestation and influence oral health. This study describes how diabetes mellitus affects oral health and oral health-related quality of life. The aim of this study was to evaluate the oral health and oral health-related quality of life of diabetic patients and compare the discriminative capability of Persian versions of two GOHAI and OHIP-14 questionnaires in these patients.

**Methods:**

A total of 350 patients with Type II diabetes mellitus, referring to the Diabetes Clinic, were selected and data were collected by GOHAI and OHIP-14 questionnaires completed by patients and clinical examinations. Oral health parameters (CAL,BI,GI,PLI,DMFT and xerostomia) were measured, also concurrent validity and conformity of two questionnaires were assessed. In order to test Discriminant analysis capabilities of two questionnaires, ADD and SC scores of questionnaires were divided into two parts and a logistic regression model was designed, which included subjective and objective variables.

**Results:**

Mean patients age was 55 years (with 75.4% female patients). The results showed that some oral conditions such as xerostomia, clinical attachment loss, number of missing teeth and plaque index were correlated to diabetes control level (HbA1c) and type of anti-diabetic medication. ADD and SC scores of two questionnaires were at high level. However, the effect of oral problems on decreasing OHRQoL was evident. Both questionnaires had acceptable concurrent validity and conformity. Moreover, there was a strong correlation between GOHAI and OHIP-14. OHIP-14 questionnaire had a higher discriminant analysis capability compared to GOHAI and better diagnosed patients who needed dental treatments: patients with higher GI, xerostomia and those wearing partial dentures.

**Conclusion:**

Diabetic patients did not show acceptable oral health status and in some extent, oral problems affected oral health-related quality of life. Psychotherapy courses and solving oral problems of the patients can improve OHRQoL. OHIP-14 had higher discriminant analysis capability and was more effective in diagnosing oral problems.

## Background

Studies have shown that diabetes is one of endocrine diseases that influence oral health of patients. Diabetes mellitus is a complex metabolic condition, which is associated with disturbances in metabolism of carbohydrates, fats and proteins [1]. Its prevalence is increasing in different parts of the world [2] and mortality resulting from this condition is due to disturbances in function of vascular system (specially microangiopathy) and a deficiency of renal function [3]. Type II diabetes mellitus is the most common type of diabetes, afflicting 95% of individuals with diabetes [4]. Changing of normal oral flora and increasing odds of infection are the results of hyperglycemia and disturbances in healing processes of injured mucous membranes due to hyposalivation, changes in salivary chemical composition, decreased immune function and changes in diet. Oral problems of diabetic patients are xerostomia and subsequent problems such as increased accumulation of plaque and calculi, candidiasis, periodontitis, periapical abscess and burning mouth syndrome, which can influence quality of life of these patients [5-7].

A large number of studies have shown that oral conditions affect economic, social and mental status of an individual. Problem such as xerostomia, edentulism, soft tissue lesions and ill-fitting prosthetic appliances influence eating habits, speech, deglutition and type of food consumed by patients and generally systemic health of patients and at the same time influence the quality of life of patients. In addition, a large number of studies have shown that oral health and general health cannot be separated from each other [8]. Questionnaires are useful tools to evaluate oral health-related quality of life. In this study Persian versions of GOHAI (GOHAI-Per) and OHIP-14 (OHIP-14-Per) were used to evaluate oral health-related quality of life (OHRQoL) [9,10]. In order to select a suitable tool for evaluation of OHRQoL its discriminant analysis properties should be determined initially [11]. Capability of questionnaires to describe OHRQoL is varied in different countries. Based on assessments made in developed countries discriminant analysis capabilities of two questionnaires are different and GOHAI has a stronger correlation with function and masticatory ability; however, OHIP-14 can predict depression. In this context, no comparisons have been made between these two questionnaires in Persian-speaking countries. Therefore, the aim of this study was to evaluate the oral health of diabetic patients and their oral health-related quality of life (OHRQoL). Also through this study psychometric properties and discriminant analysis potential of two questionnaires were compared in patient with different oral health statuses and other diabetes-dependent conditions.

## Methods

Three hundred-fifty diabetic patients who had referred to Ayatollah Rouhani Hospital in Babol, Iran, from July 2012 to March 2013 were participated in this study. The inclusion criteria was type II diabetes mellitus and the exclusion criteria was illiterate patients and subjects who were unable to fill out the questionnaires. This project was approved by Ethics and Research committee of Deputy of Research and Technology, Babol University of Medical Science. Procedural steps and the aim of the study were explained to participants initially. Duration of diabetes and demographic information including age, gender, occupation were recorded; Moreover, questions were asked about wearing removable prosthetic appliances, smoking and 9 questions about xerostomia. In xerostomia context, if 5 questions had positive answers, xerostomia was confirmed [12,13]. Data about disease, including level of the control of diabetes (HbA1c), presence of other systemic diseases, medications used and type of anti-diabetic medication used, were recorded. Besides, each patient’s opinion about their self-perceived oral and general health and their need for dental treatments were asked and recorded. Subsequently, two questionnaires related to OHRQoL, GOHAI-Per and OHIP-14-Per which were validated by Motallebnejad et al. (9) and Motallebnejad et al. (10), were completed by patients.

Finally, patients were clinically examined. DMFT, PLI and GI based on Loe and Silness method [6,14] and BI based on Barnett method [15] were measured on 28 teeth. In addition, CAL (clinical attachment loss) was measured on Ramfjord teeth.

### Scoring of GOHAI and OHIP-14 questionnaires

Reproducibility and reliability of GOHAI-Per has been confirmed. It consists of 12 questions with 5 choices, which are answered by patients. Each answer has its own score: Never = 1; Seldom = 2; Sometimes = 3; Often = 4; Always = 5.

Questions of these questionnaires were assessed oral problems during the past 3 months and three dimensions of OHRQoL (physical function, pain and discomfort and psychosocial function). All questions have negative connotations despite questions 3, 5 and 7 [9]. So except the answer of questions 3, 5 and 7, answers of other questions were reversed to achieved highest scores for good oral conditions. Total of GOHAI scores was termed ADD-GOHAI (Score of GOHAI), which had a minimum of 12 and a maximum of 60, in which a higher score indicated a higher oral health-related quality of life.

Other questionnaire which was completed by subjects was OHIP-14-Per. It consisted of 14 questions with 5 choices and each choice had its specific score: Never = 1; Seldom = 2; Sometimes = 3; Almost often = 4; In the majority of cases = 5.

In this questionnaire all questions have a negative connotation and covers seven dimensions of OHRQoL (functional limitation, physical pain, psychological discomfort, physical disability, psychological disability, social disability and handicap) [10]. So the answers of all questions were reversed to achieved highest scores for good oral conditions. Total of OHIP-14 scores was termed ADD-OHIP-14 (Additive Score of OHIP-14), which had a minimum of 14 and a maximum of 70, in which a higher score indicated a higher oral health-related quality of life.

In this study to avoid possible misunderstanding of participants 5 choices of questionnaires were placed in two groups and total of the scores were termed SC (Simple Count) Score so that if a patient chose one of two choice of “always” and “often” in GOHAI questionnaire the score of that question would be zero and if patient chose one of three choices of “sometimes”, “seldom” or “never” the score of that question would be “1”. Therefore, total of GOHAI scores (SC-GOHAI) in this method would range from zero to 12. In this system a higher score would indicate a higher oral health-related quality of life. In the same context, in OHIP-14 if a patient selected two choices of “almost often” or “in the majority of cases” score of that question would be zero and if a patient selected three choices of “sometimes”, “seldom” or “never” the score of the question would be “1” and finally total of OHIP-14 scores (SC-OHIP-14) in this method would range from zero to 14; a higher score would indicate a higher oral health-related quality of life.

### Data analysis

Statistical analyses were carried out using SPSS 18. Pearson’s correlation coefficient was used to evaluate the correlation between two quantitative variables; t-test, ANOVA and Tukey test were used to compare quantitative variables between the groups under study; and chi-squared test was used to evaluate the relationship between qualitative variables. Statistical significance was defined at P < 0.05. In order to re-evaluate the reliability of the two questionnaires whose reliability and reproducibility have already been confirmed, Cronbach’s alpha was calculated. In addition, the conformity between ADD and SC scores of each questionnaire was evaluated by calculating Pearson’s correlation coefficient. ICC (Intra-class Correlation Coefficient) was used to assess the conformity between two questionnaires. Since there are no universal criteria for OHRQoL, concurrent validity and discriminant validity were used during validation process of both questionnaires. For concurrent validity it was hypothesized that individuals with lower ADD and SC scores had lower satisfaction level with their oral status and believed that they needed dental treatment and reported low level of oral and general health; in comparison with other patients such individuals had higher CAL and DMFT. Since the scores obtained from GOHAI and OHIP-14 were not normally distributed, Mann-Whitney and Kruskal-Wallis tests were used. Evaluation of discriminant validity was carried out by comparing the GOHAI and OHIP-14 scores between groups in which oral and dental health status had been clinically evaluated and it was hypothesized that patients with a higher level of oral diseases, and lower level of oral, dental and systemic health had lower ADD and SC scores; such patients had xerostomia, had partial prosthetic appliances with only a limited number of natural teeth in oral cavity, had more lost teeth (>7), had higher PLI, GI, BI and CAL, had uncontrolled diabetes (HbA1c > 7) and had an anti diabetic medication including injection of insulin. It was also hypothesized that GOHAI and OHIP-14 scores are able to discriminate subjects with different socio-demographic characteristics, including age, sex and duration of diabetes. For continuous variables (number of lost teeth and duration of diabetes) the 50th and 75th cutoff point of their percentages were used and in order to discriminate good and bad life qualities, ADD and SC scores of two questionnaires were divided into two categories based on 25th cutoff point of their percentages. Chi-square tests were carried out and odds ratios (ORs) were calculated. Eight backward logistic regression models were designed, with P > 0.1 as criterion for elimination from the model. In the first four models all of patients and in the last four models 125 patients in which periodontal and gingival indexes were evaluated, were analyzed.

## Results

A total of 350 patients with Type II diabetes mellitus were included in the present study. The mean age of patients was 55.04 ± 10.76 years, with an age range of 22‒86 years. Patients underwent oral examinations after completing GOHAI-Per and OHIP-14-Per questionnaires. A total of 75.4% of subjects were female. Mean duration of diabetes was 8.89 ± 7.05 years and the mean of HbA1c test result was 8.13 ± 1.55, with 38.4% of subjects suffering from xerostomia. 80.2% of the subjects received cardiovascular and hypertension medications, and 22.4% took neurologic medicines and 25.8% used other drugs. 68.6% of subjects followed an oral anti-diabetic drugs and only 8.9% used tobacco; 56.6% wore no oral prosthetic appliances and 34.8% wore complete dentures. 56% of subject believed they had good oral health, with 31.7% reporting moderate and 12.3% reporting bad oral health. 32% of subjects reported proper systemic health, 50.3% reported moderate and 17.7% reported bad systemic health. Further more, 48.3% of subjects believed they required dental treatments. Means and standard deviations of PLI,GI,BI and CAL were 1.54 ± 1.13, 1.17 ±1.08,1.09 ± 1.16 and 0.69 ± 1.06, respectively. In addition, mean and standard deviation of DMFT were 13.65 ±5.55 (D:2.14 ± 2.16; M:9.34 ± 5.89; F:1.9 ± 2.6).

### Relationship between the study variables

During evaluation of the relationship between tobacco use and the variables of diabetes control (HbA1c), BI,GI, PLI, CAL, DMFT and its components showed that in patients who use tobacco, PLI increases [13(72.2%) vs. 88(43.3%); P = 0.05].

Relationship between xerostomia and HbA1c, duration of diabetes, DMFT and its components, type of anti-diabetic medication and BI, GI, PLI indices and CAL was evaluated and results showed that in patients reporting xerostomia there was a higher number of missing teeth (10.47 ± 6.22 vs. 8.73 ± 5.63; P = 0.035), with higher CAL (0.99 ± 1.43 vs. 0.52 ± 0.70; P = 0.014). In this context, patients with uncontrolled diabetes (HbA1c > 7) reported a higher prevalence of xerostomia [120(48.6%) vs. 14 (13.7%); P < 0.001]. Moreover, patients receiving an anti-diabetic medication, consisting of insulin injection, reported more xerostomia [54(49.1%) vs. 80(33.5%); P = 0.005]. Evaluation of the relationship between BI,GI,PLI indexes and CAL with the control level of diabetes showed that in patients with uncontrolled diabetes (HbA1c > 7) there was an increase in PLI and CAL [CAL:0.80 ± 1.13 vs. 0.44 ± 0.67; P = 0.028; PLI:72(51.4%) vs. 29(35.8%); P = 0.006].

Relationship between some variables such as duration of diabetes, HbA1c, DMFT and three questions about patients’ self-perceived oral health, general health and need for dental treatments was evaluated. Patients’ opinions were consistent with clinical findings, i.e. patients with higher DMFT felt a greater need for dental treatments (8.19 ± 1.16 vs. 8.06 ± 1.50; P = 0.045) and reported poor oral health (15.88 ± 6.72 vs. 13.24 ± 5.62; P = 0.019). Besides, patients with uncontrolled diabetes (HbA1c > 7) felt poor general health [49(19.8%) vs. 13(12.6%); P = 0.029].

Evaluation of relationship between type of anti-diabetic medication and BI,GI,PLI indices and CAL showed that patients receiving an anti-diabetic medication, including injection of insulin, had higher PLI and CAL (PLI:36(63.2%) vs. 65(39.6%); P = 0.022; CAL: 1.15 ± 1.55 vs. 0.52 ± 0.71; P = 0.003), with higher prevalence of uncontrolled diabetes in this group (HbA1c > 7) [91(82.7%) vs. 156(65%); P = 0.001].

### Evaluation of oral health-related quality of life

Mean scores of two GOHAI and OHIP-14 questionnaires were relatively high and ranges, means and standard deviations of ADD and SC are presented in Table 1. Correlation between ADD-GOHAI and SC-GOHAI scores was 0.896 (<0.001); correlation between ADD-OHIP-14 and SC-OHIP-14 scores was 0.873 (<0.001), which are considered high and almost similar, indicating that these parameters were consistent. Tables 2 and 3 present frequencies of subjects’ answers to each of the questions on GOHAI and OHIP-14 questionnaires.

**Table 1:** Descriptive Statistics for GOHAI-Per and OHIP-14-Per

	**ADD-GOHAI**	**ADD-OHIP-14**	**SC-GOHAI**	**SC-OHIP-14**
Range	20-60	14-70	2-12	0-14
Mean ± SD	48.71 ± 7.65	62.28 ± 9.61	9.90 ± 1.80	13.09 ± 2.05
Median	50	66	10	14
25^th^ percentile	44	60	9	13
75^th^percentile	55	68	11	14
Absence of impact	5.7%	18.9%	18.6%	67.4%

**Table 2:** Frequency distribution of the subjects’ answers to each of the questions on GOHAI-Per

	**In the past three months**	**5**	**4**	**3**	**2**	**1**
		**Never**	**Seldom**	**Sometimes**	**Often**	**Always**
	**Physical function**					
1	Limit the kind of food	230(65.7%)	41(11.7%)	35(10%)	26(7.4%)	18(5.1%)
2	Trouble biting/chewing	144(41.1%)	38(10.9%)	37(10.6%)	25(7.1%)	106(30.3%)
3	Trouble swallowing	201(57.4%)	30(8.6%)	20(5.7%)	10(2.9%)	89(25.4%)
4	Unable to speak clearly	265(75.7%)	21(6%)	26(7.4%)	13(3.7%)	25(7.1%)
	**Pain and discomfort**					
5	Discomfort when eating	163(46.6%)	48(13.7%)	41(11.7%)	33(9.4%)	65(18.6%)
8	Medications for pain	252(72%)	29(8.3%)	43(12.3%)	12(3.4%)	14(4%)
12	Sensitive teeth	208(59.4%)	31(8.9%)	53(15.1%)	28(8%)	30(8.6%)
	**Psychosocial impacts**					
6	Limit contacts with others	278(79.4%)	22(6.3%)	29(8.3%)	14(4%)	7(2%)
7	Unhappy with appearance	210(60%)	38(10.9%)	26(7.4%)	25(7.1%)	51(14.6%)
9	Worried or concerned	149(42.6%)	51(14.6%)	51(14.6%)	41(11.7%)	58(16.6%)
10	Nervous, self-conscious	262(74.9%)	26(7.4%)	40(11.4%)	14(4%)	8(2.3%)
11	Uncomfortable eating in front of others	272(77.7%)	23(6.6%)	32(9.1%)	12(3.4%)	11(3.1%)

**Table 3:** Frequency distribution of the subjects’ answers to each of the questions on OHIP-14-Per

	**In the past three months**	**5**	**4**	**3**	**2**	**1**
		**Never**	**Seldom**	**Sometimes**	**Almost Often**	**In the majority of cases**
	**Functional limitation**					
1	Trouble pronouncing words	295(84.3%)	22(6.3%)	18(5.1%)	3(0.9%)	12(3.4%)
2	Sense of taste worse	169(48.3%)	39(11.1%)	103(29.4%)	6(1.8%)	33(9.4%)
	**Physical pain**					
3	Painful aching in mouth	199(56.9%)	39(11.1%)	78(22.3%)	12(3.4%)	22(6.3%)
4	Uncomfortable to eat	225(64.3%)	48(13.7%)	50(14.3%)	11(3.1%)	16(4.6%)
	**Psychological discomfort**					
5	Self-conscious	255(72.9%)	34(9.7%)	37(10.6%)	9(2.6%)	15(4.3%)
6	Felt tense	250(71.4%)	30(8.6%)	48(13.7%)	7(2%)	15(4.3%)
	**Physical disability**					
7	Unsatisfactory diet	249(71.1%)	45(12.9%)	27(7.7%)	13(3.7%)	16(4.6%)
8	Had to interrupt meals	262(74.6%)	35(10%)	28(8%)	13(3.7%)	12(3.4%)
	**Psychological disability**					
9	Difficult to relax	241(68.9%)	49(14%)	37(10.6%)	9(2.6%)	14(4%)
10	Embarrassed	288(82.3%)	26(7.4%)	23(6.6%)	7(2%)	6(1.7%)
	**Social disability**					
11	Irritability with others	269(76.9%)	30(8.6%)	41(11.7%)	5(1.4%)	5(1.4%)
12	Difficulty doing usual jobs	300(85.7%)	23(6.6%)	18(5.1%)	5(1.4%)	4(1.1%)
	**Handicap**					
13	Felt life less satisfying	260(74.3%)	29(8.3%)	22(6.3%)	11(3.1%)	28(8%)
14	Totally unable to function	315(90%)	12(3.4%)	15(4.3%)	6(1.7%)	2(0.6%)

GOHAI-Per showed that majority of patients had a problem related to question #2: 30.3% of subjects had masticatory problems and a small percentage of patients had problems in contacting others (question#6). In fact, patients had the least problem related to this question and only 6% had answered “always” or “often”. In contrast, OHIP-14-Per showed that patients had the greatest problem related to question #13 and 11.1% of patients had answered “in the majority of cases” or “almost often” in relation to dissatisfaction with life in the past.

Both questions showed that majority of patients did not feel any problems and OHIP-14-Per showed that a higher percentage of patients were free of problems.

### The relationship between variables under study and oral health-related quality of life

Results of Concurrent validity showed that subjects with lower ADD and SC scores had poorer self-perceived oral health and systemic condition and reported a greater need for dental treatments; they also had higher CAL and DMFT (Table 4).

**Table 4:** Concurrent Validity of GOHAI-Per and OHIP-14-Per

		**n**	**GOHAI**		**OHIP-14**	
			**ADD**	**SC**	**ADD**	**SC**
	Good	196	50.38 ± 7.02	10.16 ± 1.61	64.41 ± 7.28	13.38 ± 1.44
Self-perception	Moderate	111	47.02 ± 7.16	9.73 ± 1.71	60.55 ± 10.12	12.96 ± 2.21
Of oral health	Poor	43	45.41 ± 9.59	9.09 ± 2.49	57 ± 13.98	12.09 ± 3.34
	p-value		<0.001	0.012	<0.001	0.087
	Good	112	51.33 ± 6.91	10.37 ± 1.61	65.53 ± 5.57	13.58 ± 0.93
Self-perception	Moderate	176	48.16 ± 7.07	9.88 ± 1.62	61.92 ± 9.43	13.05 ± 1.96
Of general health	Poor	62	45.53 ± 9.01	9.08 ± 2.29	57.40 ± 13.13	12.33 ± 3.23
	p-value		<0.001	<0.001	<0.001	0.014
Self-reported	Yes	169	45.79 ± 7.70	9.39 ± 1.96	59.55 ± 11.69	12.68 ± 2.70
Need for dental	No	181	51.43 ± 6.54	10.37 ± 1.50	64.82 ± 6.16	13.48 ± 1.01
Treatment	p-value		<0.001	<0.001	<0.001	0.069
AL(Attachment Loss)		125	-0.337(<0.001)	-0.331(<0.001)	-0.317(<0.001)	-0.337(0.001)
DMFT		221	-0.240(<0.001)	-0.265(<0.001)	-0.131(0.05)	-0.173(0.01)

### Comparison of GOHAI-Per and OHIP-14-Per questionnaires

**Figure 1:**
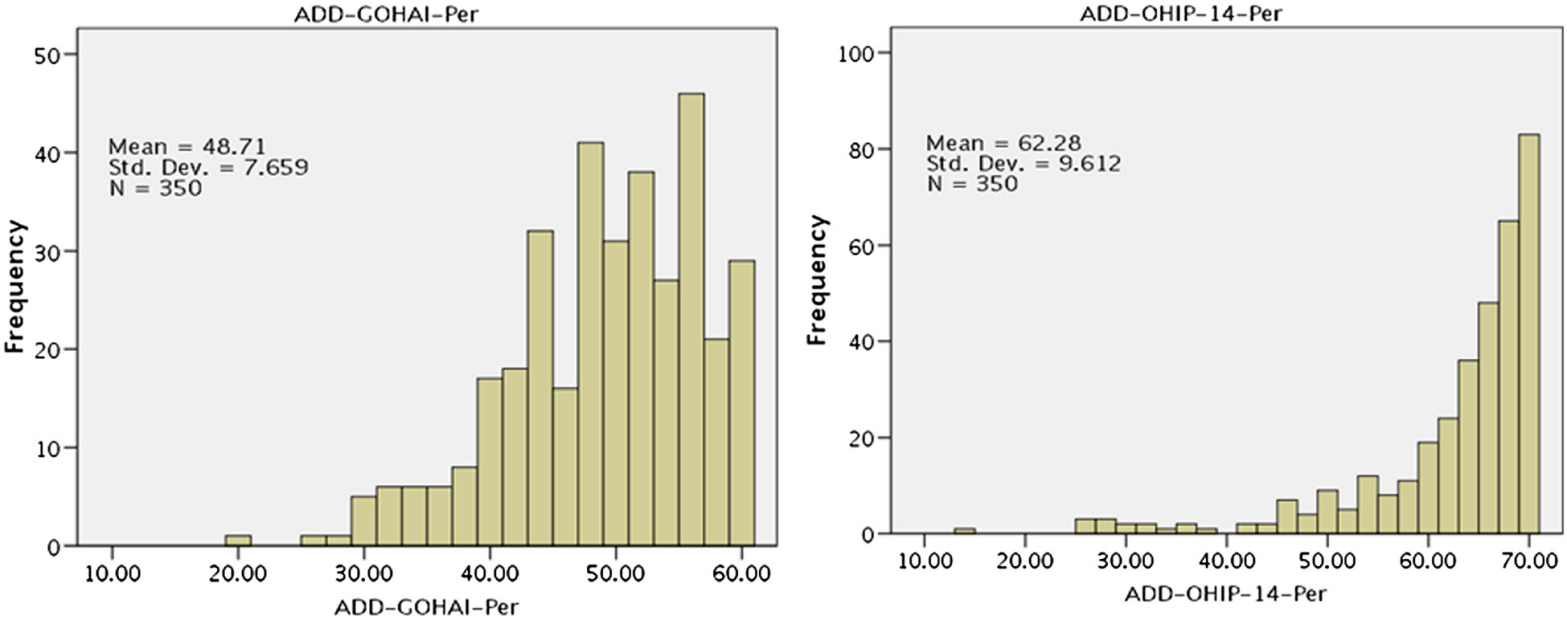
Distroibution of ADD scores in GOHAI-Per and OHIP-14-Per.

Figure 1 presents distribution of additive scores (ADD) of GOHAI and OHIP-14 questionnaires. Gradient of OHIP-14 scores graph is higher than that of OHIP-14 scores graph. The “median” of ADD-GOHAI was 50, which is less than that of ADD-OHIP-14(66). Correlation between ADD-OHIP-14 and ADD-GOHAI scores was 0.680 (<0.001); correlation between SC-OHIP-14 and SC-GOHAI scores was 0.522 (<0.001), which was considered high and almost similar, indicating that these two questionnaires are consistent with each other. Cronbach’s alpha coefficients for ADD-GOHAI and ADD-OHIP-14 were 0.68 and 0.91, respectively. ICC criteria was used to evaluate consistency between the two questionnaires, which proved acceptable (0.80).

### Disciminant validity

Evaluations showed that patients with poor health and more oral problems had lower ADD and SC scores. Use of tobacco was not entered into the model due to limited number of smoking patients (n = 18). In addition, due to limited range of D and F for classification of patients and effect of missing teeth on oral health-related quality of life of patients, among DMFT components M was entered into logistic regression models as a confounding factor. Eight models had been designed here; in the first four models 350 subjects were evaluated based on variables of age, gender, denture wearing, xerostomia, duration of diabetes, type of anti-diabetic medication, and level of diabetes control (HbA1c) (Table 5). In the second four models, 125 patients in which periodontal and gingival indexes were evaluated variables such as age, gender, denture measuring, xerostomia, duration of diabetes, type of anti-diabetic medication, level of diabetes control (HbA1c), number of missing teeth, plaque index (PLI), gingival index (GI), bleeding index (BI) and clinical attachment loss (CAL) were used for analysis (Additional file 1: Table S1).

**Table 5:** **Discrimnant validity of two questionnaires(univariate and multivariate analysis using 25**^**th **^**percentile for ADD and SC)**

		**n**	**GOHAI**		**OHIP-14**	
			**ADD<44**	**SC<9**	**ADD<60**	**SC<13**
	≤45	55	15(27.3%)	19(34.5%)	14(25.5%)	11(20%)
Age	45-65	192	61(31.8%)	68(35.4%)	56(29.2%)	36(18.8%)
	>65	103	25(24.3%)	33(32%)	24(23.3%)	16(15.5%)
	Reference		— — —	— — —	— — —	— — —
OR_Crude_(95%CI)	A_1_		1.08(0.46;2.54)	0.97(0.44;2.16)	1.15(0.47;2.81)	1.72(0.65;4.69)
	A_2_		1.37(0.74;2.53)	1.04(0.58;1.84)	1.43(0.76;2.69)	1.47(0.71;3.08)
	Reference		— — —	— — —	— — —	— — —
OR_adjusted_(95%CI)	A_1_		— — —	— — —	— — —	— — —
	A_2_		— — —	— — —	— — —	— — —
Sex	Male	86	20(23.3%)	25(29.1%)	19(22.1%)	10(11.6%)
	Female	264	81(30.7%)	95(36%)	75(28.4%)	53(20.1%)
OR_Crude_(95%CI)			1.36(0.75;2.46)	1.24(0.71;2.15)	1.20(0.65;2.21)	1.72(0.63;3.27)
OR_adjusted_(95%CI)			— — —	— — —	— — —	— — —
Xerostomia	Yes	134	41(30.6%)	53(39.6%)	46(34.3%)^ǂ^	31(23.1%)^ǂ^
	No	215	59(27.4%)	66(30.7%)	48(22.3%)^ǂ^	32(14.9%)^ǂ^
OR_Crude_(95%CI)			0.99(0.59;1.67)	0.75(0.46;1.22)	0.49(0.29;0.85)	0.61(0.33;1.12)
OR_adjusted_(95%CI)			— — —	— — —	0.49(0.29;0.84)	— — —
	Partial denture	30	12(40%)	12(40%)	12(40%)	6(20%)
Prosthesis	Complete denture	122	32(26.2%)	38(31.1%)	31(25.4%)	23(18.9%)
	With out denture	198	57(28.8%)	70(35.4%)	51(25.8%)	34(17.2%)
	Reference		— — —	— — —	— — —	— — —
OR_Crude_(95%CI)	P_1_		1.70(0.74;3.91)	1.24(0.54;2.81)	2.30(0.99;5.33)	1.39(0.51;3.82)
	P_2_		0.93(0.51;1.68)	0.77(0.44;1.36)	1.06(0.57;1.97)	1.25(0.62;2.53)
	Reference		— — —	— — —	— — —	— — —
OR_adjusted_(95%CI)	P_1_		— — —	— — —	— — —	— — —
	P_2_		— — —	— — —	— — —	— — —
type of anti-	Oral intake	240	59(24.6%)^#^	72(30%)^ǂ^	53(22.1%)^#^	32(13.3%)^#^
diabetic medication	Inject insulin	110	42(38.2%)^#^	48(43.6%)^ǂ^	41(37.3%)^#^	31(28.2%)^#^
OR_Crude_(95%CI)			2.28(1.33;3.89)	1.99(1.19;3.32)	2.44(1.40;4.26)	2.60(1.41;4.80)
OR_adjusted_(95%CI)			1.92(1.18;3.13)	1.83(1.14;2.92)	2.12(1.27;3.54)	2.39(1.36;4.20)
HbA_1C_	≤7	103	27(26.2%)	32(31.1%)	30(29.1%)	18(17.5%)
	>7	247	74(30%)	88(35.6%)	64(25.9%)	45(18.2%)
OR_Crude_(95%CI)			0.84(0.47;1.49)	0.89(0.51;1.55)	1.72(0.95;3.11)	1.43(0.72;2.85)
OR_adjusted_(95%CI)			— — —	— — —	1.82(1.02;3.25)	— — —
Diabetes	≤10	246	75(30.5%)	87(35.4%)	67(27.2%)	42(17.1%)
Duration	>10	104	26(25%)	33(31.7%)	27(26%)	21(20.2%)
OR_Crude_(95%CI)			1.82(1.01;3.26)	1.51(0.87;2.60)	1.44(0.79;2.62)	1.12(0.58;2.17)
OR_adjusted_(95%CI)			— — —	— — —	— — —	— — —

ǂ P < 0.05.

# p < 0.001.

## Discussion

Many studies have evaluated the effect of oral problems on patients with common systemic diseases and have concluded that social and psychological influences, including patient’s well-being, in quality of life [8,16-18]. Diabetic patients showed some oral problems which could associated with their OHRQoL [7,8,19]. Results of the present study showed that some oral health problems in diabetic patients are correlated with this medical condition and level of its control. In patients with low diabetic control, xerostomia was severe, and some indices under study, including. PLI and CAL were higher, consistent with results of some previous studies [7,8,19-21]. However, further studies are necessary to evaluate the relationship between xerostomia and poor control of diabetes. One of the most important complaints of diabetic patients is xerostomia, which can contribute to some oral problems, such as tooth decay, halitosis, oral burning sensation and accumulation of plaque [20-22] which can lead to gingival inflammation, in patients with poor oral hygiene; on the other hand, in diabetic patients, due to disturbances in function of white blood cells and vascular changes in gingiva, flow of nutrients to the oral tissues and removal of noxious agents from oral tissues decrease, which in turn can decrease the ability of host defense mechanisms to resist inflammation [23]. Therefore, in such patients there is higher CAL and more severe periodontal diseases. Some studies have shown a higher rate of tooth decay in diabetic patients due to xerostomia and seepage of glucose into Gingival Cervicular Fluid (GCF) [7,24]. An increase in number of lost teeth in diabetic patients with xerostomia might be attributed to tooth mobility due to periodontal diseases and an increase in the incidence of tooth decay in such patients, which is consistent with the results of present study, indicating higher CAL and more lost teeth in patients with xerostomia. Tobacco use in diabetic patients results in poor oral hygiene, increasing DMFT, PLI, GI and PDI and increases the odds of periodontitis [25,26]. This study only showed a relationshipbetween an increase in PLI and tobacco use in diabetic patients and it was not possible to establish a relationship between tobacco use and other oral signs and symptoms due to limited number of diabetic patients who used tobacco. Moreover, duration of diabetes is not correlated with prevalence and severity of periodontitis and CAL [7,27], confirmed by results of present study.

In insulin-dependent patients, duration and level of diabetes control (HbA1c > 7) were higher [8]. Control of diabetes (HbA1c) was directly correlated with type of anti-diabetic medication (oral anti-diabetic drugs or insulin included medication), and the same indices (including PLI, CAL) and xerostomia, which increased in poor control of diabetes, increased in insulin-dependent patients.

The two GOHAI-Per and OHIP-14-Per questionnaires had acceptable reproducibility and reliability, and these two questionnaires were selected to evaluate oral health-related quality of life because they are short and subjects are interested in completing them [28].

A higher percentage of middle-aged subjects, compared to the other two groups, had low OHRQoL, which might be attributed to the effect of diabetes on their mood and performance and to the fact that such patients have not been accustomed to changes which are the results and problems of old age.

In the present study, concurrent validity of two questionnaires was evaluated and confirmed. ADD and SC scores of two questionnaires were consistent with the opinions of subjects about their self-perceived oral and systemic health and need for dental treatments, also consistent with the results of previous studies in this respect [9,11]. In addition, CAL and DMFT, the effects of which on OHRQoL have been confirmed in similar studies [9,19,29], were consistent with ADD and SC scores, i.e. patients with higher CAL and DMFT had lower ADD and SC scores.

Since GOHAI and OHIP-14 have not specifically been considered predictors of clinical indicators they should be used as supplements to clinical and objective evaluations. Some studies have shown good correlation between GOHAI and OHIP-14 questionnaires and clinical observations [19,29,30] and some others have shown poor correlation between these questionnaires and clinical evaluations [9,31]. These differences are attributed to cultural factors and individuals’ living standards and their attitudes toward quality of life. Therefore, different results have been achieved in different countries with different socioeconomic conditions. Among factors which thier effect on OHRQoL was evaluated in present study, type of anti-diabetic medication, level of diabetic control (HbA1c), GI, CAL, number of teeth lost, type of denture worn by the patient and xerostomia had the ability to discriminate good OHRQoL from bad one and they can be considered factors effective in oral health-related quality of life of diabetic patients. Evaluation of various factors showed that OHIP-14-Per has a higher discriminant analysis capability compared to GOHAI-Per; however, comparison of these two questionnaires between elderly subjects in Lebanon, Canada, Germany and Japan [11,32-34] has shown a higher capability for GOHAI. This discrepancy might be attributed to content and different dimensions of two questionnaires for evaluation of OHRQoL. Good correlation was observed between GOHAI-Per and OHIP-14-Per questionnaires. However, frequency distributions of subject’ answers to questions of the questionnaires were different and OHIP-14-Per showed that a higher percentage of patients were free of problems and had more positive attitudes. In general, it can be concluded that GOHAI-Per has a more realistic view.

Although GOHAI and OHIP-14 are similar tools and both assess OHRQoL, they have different contents which can influence their ability to assess OHRQoL and the achieved results.

Also this study, showed higher ability of GOHAI in determining functional problems, pain and discomfort; on the other hand, it showed that OHIP-14 has higher ability to show psychological and social problems and physical handicaps, consistent with results of previous studies in this respect [11,33,34]. It should be pointed out that OHIP-14 can be an intermediary tool to establish a relationship between OHRQoL and well-being [35].

Based on these results, oral health affects OHRQoL more from the mental and psychological viewpoints, and functional aspects of OHRQoL are less affected in Iran.

## Conclusions

According to this study, although the diabetic patients had poor oral health, OHRQoL scores were high on both questionnaires. In fact, oral health does not very much affect oral health-related quality of life. In this context, oral health status affects psychological aspects of OHRQoL more than functional aspect. Psychotherapy courses and solving the oral problems of patients can improve OHRQoL. Besides, both GOHAI-Per and OHIP-14-Per questionnaires have proper psychometric properties and both are rather effective in determining oral health-related quality of life; however, OHIP-14-Per has a higher discriminant analysis ability. So results of present study can be used to determine oral indicators effective in oral health-related quality of life in future similar studies on OHRQoL in Iran. It is suggested compare discriminant analysis capabilities of GOHAI-Per and OHIP-14-Per.

## Abbreviations

GOHAI: Geriatric oral health assessment index; GOHAI-Per: Persian version of geriatric oral health assessment; OHIP: Oral health impact profile; OHIP-14-Per: Persian version of oral health impact profile; OHRQoL: Oral health related quality of life; DMFT: Decay, missing, filling teeth; PLI: Plaque index; GI: Gingival index; BI: Bleeding index; CAL: Clinical attachment loss; ADD-GOHAI: Additive score of geriatric oral health assessment index; ADD-OHIP: Additive score of oral oealth impact profile; SC-score: Simple count score; SC-GOHAI: Simple count geriatric oral health assessment index; SC-OHIP-14: Simple count oral health impact profile; SPSS: Statistical package software for social science; ICC: Intra-class correlation coefficient; OR: Odd ratio; GCF: Gingival cervicular fluid; PDI: Periodontal disease index.

## Competing interests

The authors declare that they have no competing interests.

## Authors’ contributions

AN carried out sampling and gathering data, participated in the design of study, preparation of manuscript. MB participated in the sampling. NJ participated in the design of study. SK performed the statistical analysis. MM proposing the idea of this study and carried out its designing. All the authors review of manuscript and approved the final manuscript.

## Supplementary Material

Additional file 1: Table S1 Discriminant validity of two questionnaires (univariate and multivariate analysis using 25th percentile for ADD and SC). * patients with Ramfjord teeth. # P < 0.001. ǂ P < 0.05. Click here for file

## References

[B1] Hoseinpour Jajarm H Mohtasham N Rangiani A Evaluation of oral mucosa epithelium in type II diabetic patients by an exfoliative cytology method Oral Sciences 2008 50 355 40 10.2334/josnusd.50.335 18818471

[B2] Powers AC Fauci AS, Braunwald E, Kasper DL, Hauser SL, Longo DL, Jameson JL, Loscalzo J Diabetes Mellitus Harrisons Principle Of Internal Medicine, Volume 2 2008 17 United states of America: McGraw-Hil 2276 2277 2292 – 2293

[B3] Shareef BT Ang KT Naik VR Qualitative and quantitative exfoliative cytology of normal oral mucosa in type 2 diabetic patients Med Oral Pathol Oral cir Bucal 2008 13 693 696 18978708

[B4] Kuzuya T Nakagawa S Satoh J Kanazawa Y Iwamoto Y Kobayashi M Nanjo K Sasaki A Seino Y Ito C Shima K Nonaka K Kadowaki T Report of the committee on the classification and diagnostic criteria of diabetes mellitus Diabetes Res Clin Pract 2002 55 65 85 10.1016/S0168-8227(01)00365-5 11755481

[B5] Little JW Falace DA Miller CS Rhodis NL Dental Management Of The Medically Compromised Patient 2002 ST Louis: Mosby

[B6] Newman MG Takei HH Klol levold PR Carranza FA Carranza’S Clinical Periodontology 2006 China: Saunders

[B7] Sandberg GE Sundberg HE Fjellstrom CA Wikblad KF Type 2 diabetes and oral health : a comporison between diabetic and non- diabetic Subjects Diabetes Res Clin Pract 2000 50 27 34 10936666 10.1016/s0168-8227(00)00159-5

[B8] Sardberg GE Wikblod KF Oral health and health – related quality Of life in type 2 diabetic patients and non – diabetic controls Acta Odontal Scand 2003 61 141 148 10.1080/00016350310002559 12868687

[B9] Motallebnejad M Mottaghi K Mehdizade S Alaedini F Bijani A Reliability and validity of the Persian version of the general oral health assessment index (GOHAI) Caspian J Dent Res 2012- 2013 1 8 17

[B10] Motallebnejad M Hadian H Mehdizade S Hajiahmadi M Validity and reliability of the Persian version of the oral health impact profile (OHIP)-14 Caspian J Intern Med 2011 2 314 320 24551438 PMC3895829

[B11] EL Osta N Tubert-Jeannin S Hennequin M Bou Abboud Naaman N EL Osta L Geahchan N Comparison of the OHIP-14 and GOHAI as measures of oral health among elderly in Lebanon Health Qual life outcomes 2012 10 31 doi: 10.1186/1477-7525-10-131 10.1186/1477-7525-10-31 23110518 PMC3495839

[B12] Seob JS Chang Chung S Hong KH Young KU Chung JW Dry mouth among the elderly in Korea: a survey of prevalence, severity, and associated factors Oral Surg Oral Med Oral Pathol Oral Radiol Endod 2010 110 403 475 10.1016/j.tripleo.2010.05.004 20868994

[B13] Torres SR Peixoto CB Coldas DM Silva EB Akiti T Nucci M de Uzeda M Relationship between salivary flow rates and condida counts in subjects with xerostomia Oral Surg Oral Med Oral Path Oral Radial Endod 2002 93 144 150 10.1067/moe.2002.120805 11862202

[B14] Madhusudan K Pralhad L Dasar: Principles and Practice of Public Health Dentistry 2010 New Delhi: Jaypee Brothers

[B15] Augusta Bessa Rebelo M Corrêa de Queiroz A Panagakos FS, Davies RM Gingival Indices: State of Art Gingival Diseases - Their Aetiology, Prevention and Treatmen 2011 1 Croatia: InTech 44 45

[B16] Locker D Health outcomes of oral disorders[abstract] Int J Epidemiol 1995 24 Suppl 1 s85 s89 7558559 10.1093/ije/24.supplement_1.s85

[B17] Slade GD Assessing change in quality of life using the oral health impact profile Community Dent Oral Epidemiol 1998 26 52 61 10.1111/j.1600-0528.1998.tb02084.x 9511843

[B18] Gift HC Quality of life–an outcome of oral health care? J Public Health Dent 1996 56 67 68 10.1111/j.1752-7325.1996.tb02398.x 8863288

[B19] Drumond-Santana T Costa FO Zenóbio EG Soares R Santana TD Impact of periodontal disease on quality of life for dentate diabetics Cad Saude Publica 2007 23 637 644 10.1590/S0102-311X2007000300022 17334577

[B20] Manfredi M McCullough MJ Vescovi P Al-Kaarawi ZM Porter SR Update on diabetes mellitus and related oral diseases Oral Dis 2004 10 187 200 10.1111/j.1601-0825.2004.01019.x 15196139

[B21] Vernillo AT Dental considerations for the treatment of patients with diabetes mellitus J Am Dent Assoc 2003 134 245 335 10.14219/jada.archive.2003.0366 18196670

[B22] Lux J BA MSW Review of the oral disease-systemic disease link. Part I: heart disease, diabetes CJDH 2006 40 288 342

[B23] Ueta E Osaki T Yoneda K Yamamoto T Prevalence of diabetes mellitus in odontogenic infections and oral candidiasis: an analysis of neutrophil suppression J Oral Pathol Med 1993 22 168 174 10.1111/j.1600-0714.1993.tb01051.x 8391079

[B24] Hallmon WW Mealey BL Implications of diabetes mellitus and periodontal disease Diabetes Educ 1992 18 310 315 10.1177/014572179201800409 1628532

[B25] Sharma R Sunder Raj S Vinod K Reddy YG Vela D Bailoor D Comparison of oral health indicators in type 2 diabetes mellitus patients and controls J Indian Acad Oral Med Radiol 2011 23 168 172

[B26] Obradović R Kesić LJ Gašić J Petrović M Živković N Role of smoking in periodontal disease among diabetic patients West Indian Med J 2012 61 98 101 22808575

[B27] Soskolne WA Epidemiological and clinical aspects of periodontal diseases in diabetics Ann Periodontol 1998 3 3 12 10.1902/annals.1998.3.1.3 9722685

[B28] Hebling E Pereira AC Oral health-related quality of life: a critical appraisal of assessment tools used in elderly people Gerodontology 2007 24 151 161 10.1111/j.1741-2358.2007.00178.x 17696892

[B29] Li Z Zhu L Sha Y Effects of periodontal health and related factors on the oral health – related quality of life in type 2 diabetic patients with chronic periodontitis[abstract] Hua Xi Kou Qiang Yi Xue Za Zhi 2011 29 s379 21932658

[B30] de Pinho AM S Borges CM de MH NG˜aA Ferreira e Ferreira F Duarte Vargas AM Impact of periodontal disease on the quality of life of diabetics based on different clinical diagnostic criteria Int J Clin Dent 2012 doi:10.1155/2012/986412 10.1155/2012/986412 PMC3465991 23056051

[B31] Ghaem H Borhani Haghighi A Zeighami B Dehghan A Validity and reliability of the Persian version of the Parkinson disease quality of life (PDQL) questionnaire Journal of Kerman University of Meical Sciences 2010 17 49 58

[B32] Hassel AJ Steuker B Rolko C Keller L Rammelsberg P Nitschke I Oral health-related quality of life of elderly Germans–comparison of GOHAI and OHIP-14 Community Dent Health 2010 27 242 7 21473361

[B33] Ikebe K Hazeyama T Enoki K Murai S Okada T Kagawa R Matsuda K Maeda Y Comparison of GOHAI and OHIP-14 measures in relation to objective values of oral function in elderly Japanese Community Dent Oral Epidemiol 2012 40 406 414 doi:10.1111/j.1600-0528.2012.00683.x. Epub 2012 Mar 31 10.1111/j.1600-0528.2012.00683.x 22469135

[B34] Locker D Matear D Stephens M Lawrence H Payne B Comparison of the GOHAI and OHIP-14 as measures of the oral health-related quality of life of the elderly Community Dent Oral Epidemiol 2001 29 373 81 10.1034/j.1600-0528.2001.290507.x 11553110

[B35] Hassel AJ Danner D Schmitt M Nitschke I Rammelsberg P Wahl HW Oral health-related quality of life is linked with subjective well-being and depression in early old age Clin Oral Investig 2011 15 691 697 doi:10.1007/s00784-010-0437-3. Epub 2010 Jun 26 10.1007/s00784-010-0437-3 20582443

