# Supplementary material for: Oral health-related quality of life in diabetic patients: comparison of the Persian version of Geriatric Oral Health Assessment Index and Oral Health Impact Profile: A descriptive-analytic study
Source: J Diabetes Metab Disord. 2014 Feb 4;13:32. doi: 10.1186/2251-6581-13-32 (PMC4015305; doi:10.1186/2251-6581-13-32)
Supplement: Additional file 1: Table S1 — Discriminant validity of two questionnaires (univariate and multivariate analysis using 25th percentile for ADD and SC). * patients with Ramfjord teeth. # P < 0.001. ǂ P < 0.05. [file 2251-6581-13-32-S1.pdf]

Table S1 - Discriminant validity of two questionnaires(univariate and multivariate analysis using 25th percentile for ADD and SC)

		n=125[*]	GOHAI		OHIP-14	
			ADD<44	SC<9	ADD<60	SC<13
Age	≤45	40	11(27.5%)	13(32.5%)	11(27.5%)	8(20%)
	45-65	74	22(29.7%)	22(29.7%)	23(31.1%)	10(13.5%)
	>65	11	3(27.3%)	4(36.4%)	3(27.3%)	3(27.3%)
OR_{Crude}(95%CI)	Reference		-----	-----	-----	-----
	A_1		0.89(0.13;5.97)	0.86(0.14;5.39)	0.87(0.09;8.18)	0.25(0.02;2.52)
	A_2		1(0.18;5.51)	0.63(0.12;3.35)	1.11(0.14;8.77)	0.13(0.01;1.18)
$OR_{adjusted}$(95%CI)	Reference		-----	-----	-----	-----
	A_1		-----	-----	-----	-----
	A_2		-----	-----	-----	-----
Sex	Male	39	9(23.1%)	10(25.6%)	10(25.6%)	5(12.8%)
	Female	86	27(31.4%)	30(34.9%)	28(32.5%)	16(18.6%)
OR_{Crude}(95%CI)			1.53(0.54;4.33)	1.77(0.61;5.13)	1.22(0.39;3.80)	1.49(0.37;5.95)
$OR_{adjusted}$(95%CI)			-----	-----	-----	-----
Xerostomia	Yes	36	12(33.3%)	14(38.9%)	17(47.2%)[#]	9(25%)
	No	89	23(25.6%)	25(28.8%)	20(22.5%)[#]	11(12.3%)
OR_{Crude}(95%CI)			0.72(0.23;2.27)	0.46(0.15;1.47)	0.11(0.03;0.40)	0.38(0.09;1.61)
$OR_{adjusted}$(95%CI)			-----	-----	0.15(0.05;0.45)	-----
Prosthesis	Partial denture	10	5(50%)	6(60%)	5(50%)	3(30%)
	Complete denture	5	2(40%)	1(20%)	2(40%)	1(20%)
	With out denture	110	29(26.4%)	33(30%)	30(27.3%)	17(15.5%)
OR_{Crude}(95%CI)	Reference		-----	-----	-----	-----
	P_1		3.13(0.59;16.56)	3.44(0.59;20.06)	7.48(1.07;52.10)	2.37(0.25;22.60)
	P_2		1.23(0.14;10.51)	0.31(0.02;4.25)	1.83(0.13;24.67)	0.17(0.00;7.63)
$OR_{adjusted}$(95%CI)	Reference		-----	-----	-----	-----
	P_1		-----	-----	7.28(1.56;33.96)	-----
	P_2		-----	-----	1.39(0.17;11.48)	-----
type of anti-diabetic medication	Oral intake	98	23(23.5%)[ǂ]	27(27.5%)	25(25.5%)	12(12.2%)[ǂ]
	Inject insulin	27	12(44.4%)[ǂ]	12(44.4%)	12(44.4%)	8(29.6%)[ǂ]
OR_{Crude}(95%CI)			2.42(0.82;7.13)	1.73(0.57;5.21)	2.18(0.68;7.04)	2.54(0.69;9.37)
$OR_{adjusted}$(95%CI)			2.57(1.05;6.28)	-----	-----	-----
HbA_{1C}	≤7	50	13(26%)	15(30%)	16(32%)	8(16%)
	>7	75	22(29.3%)	24(32%)	21(28%)	12(16%)
OR_{Crude}(95%CI)			0.96(0.34;2.66)	1.11(0.39;3.14)	3.83(1.17;12.54)	1.58(0.41;6.03)
$OR_{adjusted}$(95%CI)			-----	-----	3.18(1.14;8.90)	-----
Diabetes Duration	≤10	97	29(29.9%)	33(34%)	29(29.9%)	15(15.5%)
	>10	28	7(25%)	7(25%)	9(32.1%)	5(17.9%)
OR_{Crude}(95%CI)			2.03(0.51;7.96)	2.80(0.69;11.34)	1.62(0.41;6.33)	3.29(0.55;19.85)
$OR_{adjusted}$(95%CI)			-----	-----	-----	-----
Missing Teeth	≤7	81	20(24.7%)	19(23.5%)[#]	22(27.2%)	12(14.8%)
	>7	44	16(36.4%)	21(47.7%)[#]	16(36.4%)	9(20.4%)
OR_{Crude}(95%CI)			0.70(0.26;1.85)	0.33(0.13;0.86)	1.03(0.35;3.02)	1.27(0.35;4.66)
$OR_{adjusted}$(95%CI)			-----	0.35(0.16;0.77)	-----	-----
PLI (plaque index)	Excellent	29	8(27.6%)	7(24.1%)	9(31%)	5(17.2%)
	Good	1	0(0%)	0(0%)	1(100%)	0(0%)
	Fair	45	11(24.4%)	14(31.1%)	9(20%)	5(11.1%)
	Poor	50	17(34%)	19(38%)	19(38%)	11(22%)
OR_{Crude}(95%CI)	Reference		-----	-----	-----	-----
	PLI1		0.82(0.19;3.49)	0.39(0.09;1.80)	1.55(0.31;7.75)	3.13(0.39;25.02)
	PLI2		0.00	0.00	0.00	0.00
	PL3		0.46(0.13;1.57)	0.37(0.11;1.24)	0.28(0.08;1.01)	0.43(0.08;2.32)

OR$_{adjusted}$(95%CI)	Reference		-----	-----	-----	-----
	PLI1		-----	-----	-----	-----
	PLI2		-----	-----	-----	-----
	PL3		-----	-----	-----	-----
GI (gingival index)	Mild Gingivitis	89	22(24.7%)	25(28.1%)	22(24.7%)	11(12.4%)
	Moderate Gingivitis	17	5(29.4%)	5(29.4%)	6(35.3%)	4(23.5%)
	Severe Gingivitis	19	9(47.4%)	9(47.4%)	10(52.6%)	6(31.6%)
OR$_{Crude}$(95%CI)	Reference		-----	-----	-----	-----
	GI1		0.12(0.01;1.01)	0.09(0.01;0.82)	0.13(0.02;0.96)	0.16(0.01;1.82)
	GI2		0.17(0.02;1.49)	0.12(0.01;1.18)	0.14(0.02;1.12)	0.21(0.02;2.45)
OR$_{adjusted}$(95%CI)	Reference		-----	-----	-----	-----
	GI1		-----	-----	0.19(0.06;0.63)	-----
	GI2		-----	-----	0.18(0.04;0.88)	-----
BI (bleeding index)	0	57	17(29.8%)	18(31.6%)	13(22.8%)	6(10.5%)
	0.1- 0.9	25	8(32%)	11(44%)	11(44%)	7(28%)
	1-1.9	22	3(13.6%)	4(18.2%)	5(22.7%)	2(9.5%)
	2-3	21	8(38.1%)	7(33.3%)	9(42.9%)	5(23.8%)
OR$_{Crude}$(95%CI)	Reference		-----	-----	-----	-----
	BI1		6.29(0.62;63.84)	13.79(1.22;155.87)	1.14(0.13;9.84)	1.30(0.09;19.51)
	BI2		4.24(0.49;36.79)	10.91(1.12;106.10)	2.75(0.37;20.34)	3.86(0.37;40.29)
	BI3		0.52(0.06;4.13)	1.20(0.15;9.27)	0.73(0.11;4.81)	0.77(0.07;7.91)
OR$_{adjusted}$(95%CI)	Reference		-----	-----	-----	-----
	BI1		-----	-----	-----	-----
	BI2		-----	-----	-----	-----
	BI3		-----	-----	-----	-----
CAL(Clinical Attachment Loss)		125	0.94±1.34[ǂ]	0.89±1.29[ǂ]	0.91±1.42	1.24±1.71[#]
OR$_{Crude}$(95%CI)			1.35(0.72;2.52)	1.34(0.69;2.59)	0.96(0.51;1.79)	2.16(0.91;5.13)
OR$_{adjusted}$(95%CI)			-----	-----	-----	1.76(1.14;2.73)

* patients with Ramfjord teeth

[#] $P < 0.001$

[ǂ] $P < 0.05$
